# The complete mitochondrial genome of *Membranipora villosa* Hincks, 1880 (Bryozoa: Gymnolaemata: Cheilostomatida): phylogenetic relationship of two kelp-encrusting bryozoans within the suborder Membraniporina

**DOI:** 10.1080/23802359.2024.2364755

**Published:** 2024-06-18

**Authors:** Geon Woo Noh, Sang-Hwa Lee, Hyun Sook Chae, Ho Jin Yang, Hyun Il Yoo, Ji Eun Seo

**Affiliations:** a Department of Food Science and Biotechnology, Graduate School, Woosuk University, Wanju, Republic of Korea; b Invertebrate Diversity Institute (InDI), Cheongju, Republic of Korea; c Department of Life Science, Woosuk University, Jincheon, Republic of Korea; d Aquatic Plant Variety Center, NIFS, Mokpo, Republic of Korea

**Keywords:** *Membranipora villosa*, *Membranipora membranacea*, mitogenome, phylogeny, Membraniporina

## Abstract

The two commonest kelp-encrusting bryozoans, *Membranipora villosa* and *M. membranacea*, are difficult to distinguish morphologically. Molecular studies of *M. villosa* should thus be helpful for the identification of both species because the mitogenome of *M. membranacea* was already sequenced. The complete mitogenome of *M. villosa* collected from Sinjido was determined in this study through Illumina NovaSeq sequencing. Maximum-likelihood (ML) analysis was based on concatenated 13 protein-coding genes dataset from nine bryozoan species. The mitogenome length was 15,407 bp, and its gene arrangement was similar to those of the mitogenome of other membraniporids, having 13 PCGs, two ribosomal RNAs, and 22 tRNAs. It had an overall A + T content of 63.7% (29.7% A, 16.7% C, 19.6% G, and 34.0% T). *M. villosa* and *M. membranacea* showed sequence differences of 20% for the total length of mitogenome and 16.1.% for 13 PCGs. Molecular data definitely consider them to be separate species. Phylogenetic analyses based on the amino acids of 13 PCGs indicated that *M. villosa* has the closest relationship with another kelp-encrusting bryozoan, *M. membranacea* of membraniporids. The phylogenetic position of genera and families within the suborder Membraniporina coincides with the Bayesian phylogenetic analysis of the mixed concatenated alignment consisting of three partitions.

## Introduction

**Figure 1.**
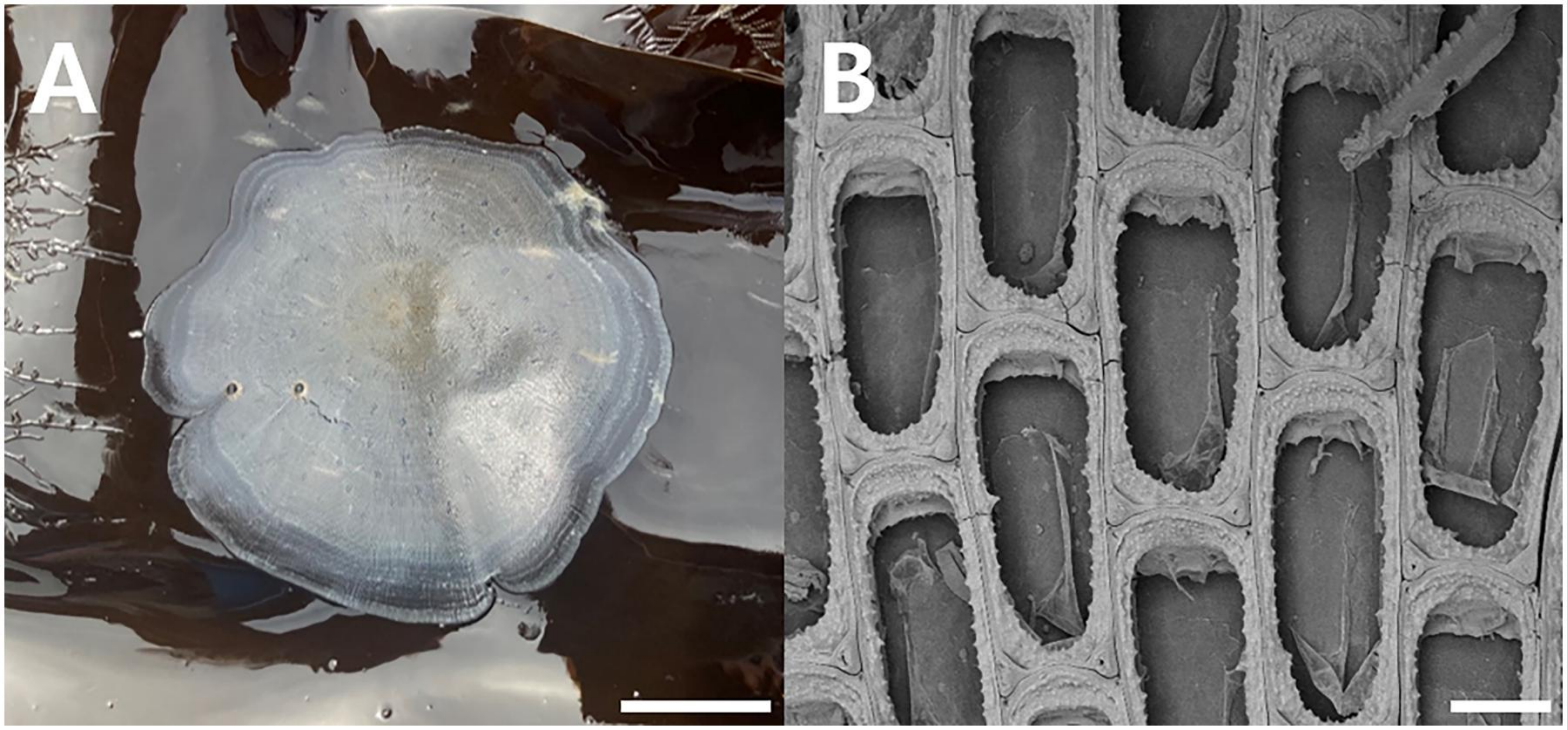
*Membranipora villosa*. (A) Colony encrusting *Saccharina japonica,* from kelp farm. (B) Autozooids, bleached, showing serrated lateral borders of cryptocyst and a short, blunt, hollow, spine-like knob at each proximal corner, SEM image, scale bar:1cm (a), 100㎛ (B). Photographs were taken by Geon Woo Noh (SEM image) and Ji Eun Seo (colony).

Two nominal common kelp-encrusting species of membraniporids belonging to the phylum Bryozoa, *Membranipora villosa* Hincks, 1880 (Bryozoa: Cheilostomatida: Membraniporina: Membraniporidae) from the North Pacific including Korean waters and *Membranipora membranacea* (Linnaeus, 1767) from mainly the Atlantic, are known. Of the two, *M. villosa* is the most predominant epialgal organism in Korean kelp farms (Kim et al. 2017) (Figure 1(A)); *M. membranacea* sensu stricto has yet to be found in Korean waters. As currently understood, *M. villosa* typically has a moderately to coarsely serrated cryptocyst and cuticular spines on the frontal membrane, whereas *M. membranacea* is normally characterized by a thinner lateral cryptocyst that is nearly smooth or only weakly denticulate and there are no frontal cuticular spines (Dick et al. 2005; Seo 2010) (Figure 1(B)). Both species have a short, blunt, hollow, conical, spine-like knob at each proximal corner. The two species, if such they are, are so morphologically similar that several studies have sought to distinguish them (Yoshioka 1982; Soule et al. 1995; Schwaninger 1999; Dick et al. 2005). In addition, Korean *Membranipora* used in a biochemical study was taken to be *M. membranacea* and unaccompanied by any morphological description (Getachew et al. 2014), highlighting the confusion between these two nominal species.

The complete mitochondrial genome of *M. membranacea* from the Atlantic Ocean has been sequenced (GenBank Accession No.: OU612079), while no sequencing was conducted with *M. villos*a. Previous molecular studies have focused on the overall phylogenetic analysis of bryozoans (Orr et al. 2020, 2021; Grant et al. 2023) or the phylogenetic relationships between bryozoans and other animals (Jang and Hwang 2009; Shen et al. 2012; Gim et al. 2018). In this study, to distinguish the morphologically similar species, we describe the complete mitochondrial genome of *M. villosa* through Illumina NovaSeq sequencing for the first time. The phylogenetic relationships of the families and genera within the suborder Membraniporina are also analyzed herein.

## Materials and methods

The specimen was collected from the subtidal zone of *Saccharina japonica* (Areschoug) C.E.Lane, C.Mayes, Druehl & G.W. Saunders, 2006 farm, Sinji-do, Wando-gun, Jeollanamdo, Republic of Korea (34°20′28.12″ N, 126°54′06.79″ E). The voucher specimen was deposited at the Marine Bryozoa Bank of Korea, Woosuk University, Jincheon (voucher number: MBRBK-PS-006, Prof. Ji Eun Seo, jeseo@woosuk.ac.kr). One live specimen of *M. villosa* was used for this study.

The mt DNA enrichment procedure is divided into three steps (mitochondria isolation, mt DNA extraction, and mt DNA amplification). First, the mitochondria were isolated using the Qproteome Mitochondrial Isolation Kit (Qiagen Co., Hilden, Germany), and mt DNA was extracted using an E.Z.N.A Mollusc DNA Kit (Omega Co., Norcross, GA). The REPLI-g Mitochondrial DNA Kit (Qiagen Co., Hilden, Germany) was used for amplification. Amplified mt DNA of *M. villosa* was sequenced using the Illumina NovaSeq platform at NICEM (Seoul, South Korea). Short-read DNA sequences were assembled and analyzed by NOVOplasty v.4.3.3 (Dierckxsens et al. 2017) and Geneious 9.1.8 (Biomatters Ltd, Auckland, New Zealand) using *M. membranacea* mitogenome data as a reference sequence. Of nine species used in this study, comprising six Membraniporina species and two bugulids (outgroup), the annotated genes of *B. grandicella* (Canu & Bassler, 1929), *B. neritina* (Linnaeus, 1758), and *M. membranacea* were available from the GenBank. The authors annotated the remaining six species, including *M. villosa*, using MITOS Web Server (Bernt et al. 2013).

The mitogenome sequences (13 protein-coding genes as amino acids, excluding stop codon) of nine species, were aligned using the multiple sequence alignment program, MAFFT v.7 (Katoh and Standley 2013) and were concatenated for phylogenetic analysis. PartitionFinder v. 2.1.0 (Lanfear et al. 2012) was used to find the best partition scheme and the best-fit model for amino acids sequence alignments. A phylogenetic tree was reconstructed based on the bryozoan concatenated dataset using the maximum-likelihood (ML) method with the GTR + G + I model in raxmlGUI 2.0 (Edler et al. 2021), and the bootstrap values were calculated from 10,000 replicates.

## Results

**Figure 2.**
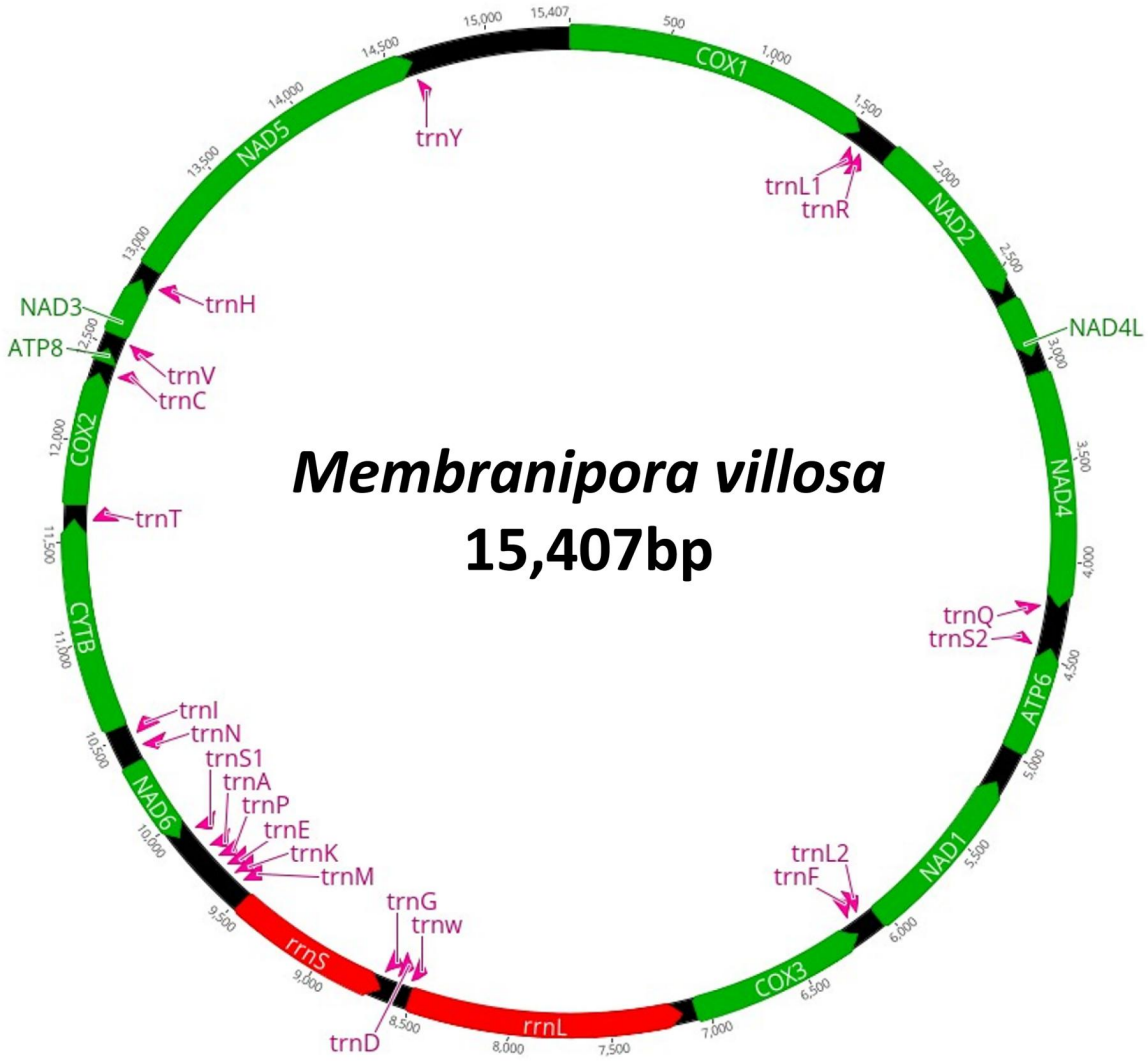
Circular map of the mitogenome of *M. villosa.*

The complete mitogenome of *M. villosa* was found to be 15,407 bp in length (Figure 2). The overall base composition was 36.3% of A, 34.0% of T, 16.7% of C, and 19.6% of G, showing a slight A–T bias (63.7%). The genome contained full complements of 13 PCGs, two ribosomal RNA genes, and 22 tRNAs. The heavy strand (H-strand) encodes *atp8*, *cox1-2*, *cytb*, *nad2-5*, *nad4L, trnC*, *trnH*, *trnI, trnL1*, *trnQ*, *trnR*, *trnT*, and *trnY*. The light strand (L-strand) encodes the remaining protein-coding genes (*atp6*, *cox3*, *nad1*, and *nad6*), the two rRNA genes (*rrnL* and *rrnS*), and 14 tRNAs (*trn*A, *trnD*, *trnE*, *trnF*, *trnG*, *trnK*, *trnL2*, *trnM*, *trnN*, *trnP*, *trnS1*, *trnS2*, *trnV*, and *trnW*). All PCGs began with ATG or ATA as a start codon, and nine genes (*atp8*, *cox1*-*2*, *cytb*, *nad1-3*, *nad5*, and *nad6*) and four genes (*atp6*, *cox3*, *nad4*, and *nad4L*) use TAG and TAA as a stop codon, respectively.

**Figure 3.**
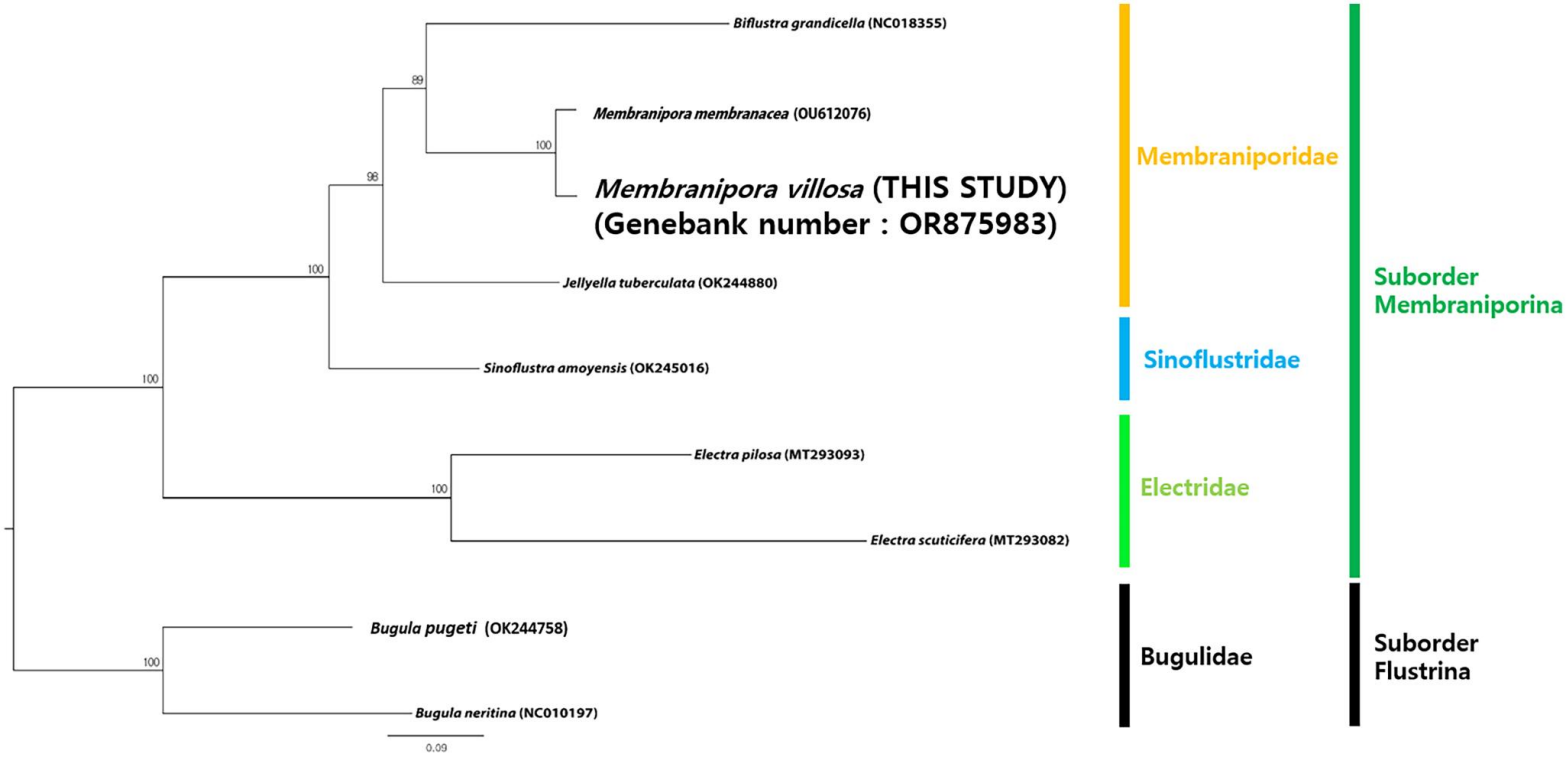
Maximum-likelihood tree based on concatenated 13 PCGs sequence dataset from nine bryozoan species. Two bugulid species were used as outgroups. GenBank accession numbers of each sequence were marked behind their corresponding species names in the tree. The following sequences were used: *Biflustra grandicella* (NC018355; Shen et al. 2012), *Membranipora membranacea* (OU612076; Grant et al. 2023), *Jellyella tuberculata* (OK244880; reference not available), *Sinoflustra amoyensis* (OK245016; reference not available), *Electra pilosa* (MT293093; reference not available), *Electra scuticifera* (MT293082; reference not available), *Bugula pugeti* (OK244758; reference not available) and *Bugula neritina* (NC010197; Jang and Hwang 2009).

The ML tree (Figure 3) in the current study showed that *M. villosa* nests within the genus *Membranipora* and has a close relationship with *M. membranacea* with very high nodal support (100% BP in ML). The Membraniporidae is fully supported as a monophyletic family in our phylogenetic analysis (100% BP in ML) and the suborder Membraniporina forms two clades: Electridae is not clustered with the other two families, Membraniporidae and Sinoflustridae.

## Discussion and conclusions

Alpha-level taxonomy in the bryozoan order Cheilostomatida relies almost exclusively on hard-part morphology (Dick and Mawatari 2005). Two morphologically similar species of the genus *Membranipora* show a preference for kelp. *M. villosa* is a major kelp-encrusting bryozoan in Japan, Hong Kong, and the northeastern Pacific Ocean, including Korean waters, settling on such species as *Saccharina japonica* (Osburn 1950; Huang et al. 1986; Dick et al. 2005; Chae and Seo 2019), whereas *M. membranacea* mainly occurs in the Atlantic Ocean (Hayward and Ryland 1998; Saunders and Metaxas 2008). *M. villosa* is nominally distinct but, owing to close similarity with *M. membranacea*, its status has been questioned. In this study, *M. villosa* and *M. membranacea* showed sequence differences of 20% for the total length of mitogenome and 16.1% for 13 PCGs. Molecular data definitely consider them to be separate species.

A ML tree based on a concatenated 13 PCGs sequence dataset from nine bryozoan species in the current study shows that *M. villosa* and *M. membranacea* have the closest relationship with very high nodal support (100% BP in ML). Interestingly, the two species having close molecular relationships show morphological and ecological similarities.

A phylogenetic analysis of the mitochondrial genome sequence of *M. villosa* was also conducted to determine the phylogenetic relationships within the suborder Membraniporina. Three genera belonging to Membraniporidae are in the same position in the phylogenetic tree as the previous studies (Hao et al. 2005; Orr et al. 2020; Grant et al. 2023). Electridae, one clade within the suborder Membraniporina, has a sister group relationship to the other clade, comprising Membraniporidae and Sinoflustridae, showing similar results to previous studies (Hao et al. 2005; Orr et al. 2020; Grant et al. 2023). The phylogenetic tree also shows that Sinoflustridae is not nested in Membraniporidae or Electridae, and this result coincides with the fact that Gordon (2009) proposed a new family with morphological data and Bayesian phylogenetic analysis of the mixed concatenated alignment consisting of three partitions (Grant et al. 2023).

## Supplementary Material

Supplemental Material

## Data Availability

The data supporting this study’s findings are available in the GenBank of NCBI at https://www.ncbi.nlm.nih.gov under the accession OR875983. The associated BioProject, SRA, and Bio-sample numbers are PRJNA1041575, SRX22552200, and SAMN38288304, respectively.
