# Supplementary figures and images for: The complete mitochondrial genome of Membranipora villosa Hincks, 1880 (Bryozoa: Gymnolaemata: Cheilostomatida): phylogenetic relationship of two kelp-encrusting bryozoans within the suborder Membraniporina
Source: Mitochondrial DNA B Resour. 2024 Jun 18;9(6):782–6. doi: 10.1080/23802359.2024.2364755 (PMC11188949; doi:10.1080/23802359.2024.2364755)

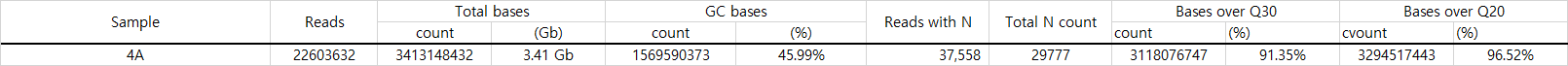

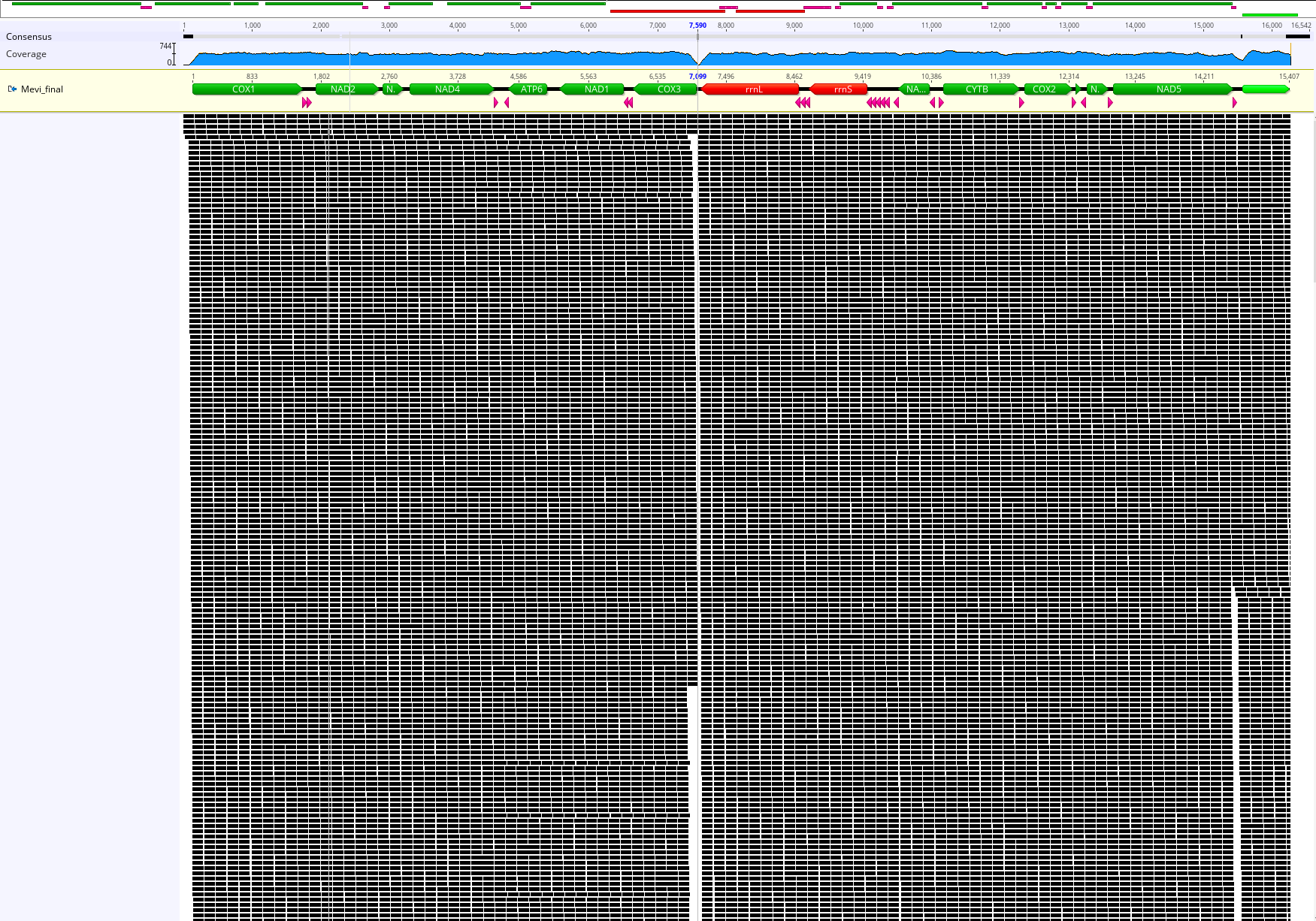

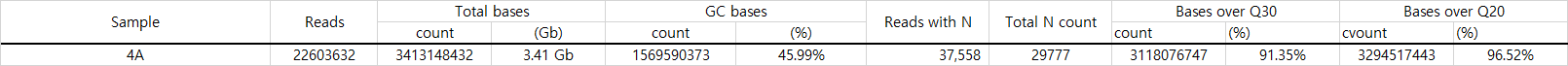

Supplement: Supplemental Material [file TMDN_A_2364755_SM9902.docx]
